# Assessment of *Bifidobacterium* Species Using *groEL* Gene on the Basis of Illumina MiSeq High-Throughput Sequencing

**DOI:** 10.3390/genes8110336

**Published:** 2017-11-21

**Authors:** Lujun Hu, Wenwei Lu, Linlin Wang, Mingluo Pan, Hao Zhang, Jianxin Zhao, Wei Chen

**Affiliations:** 1 State Key Laboratory of Food Science and Technology, Jiangnan University, Wuxi 214122, China; 7130112038@vip.jiangnan.edu.cn (L.H.); luwenwei@jiangnan.edu.cn (W.L.); wanglllynn09@163.com (L.W.); mingluopan@163.com (M.P.); zhanghao@jiangnan.edu.cn (H.Z.); chenwei66@jiangnan.edu.cn (W.C.); 2 School of Food Science and Technology, Jiangnan University, Wuxi 214122, China; 3 National Engineering Research Center for Functional Food, Jiangnan University, Wuxi 214122, China; 4 Beijing Innovation Centre of Food Nutrition and Human Health, Beijing Technology and Business University, Beijing 100048, China

**Keywords:** *Bifidobacterium*, *groEL*, species level, biodiversity, MiSeq high-throughput sequencing

## Abstract

The next-generation high-throughput sequencing techniques have introduced a new way to assess the gut’s microbial diversity on the basis of 16S rRNA gene-based microbiota analysis. However, the precise appraisal of the biodiversity of *Bifidobacterium* species within the gut remains a challenging task because of the limited resolving power of the 16S rRNA gene in different species. The *groEL* gene, a protein-coding gene, evolves quickly and thus is useful for differentiating bifidobacteria. Here, we designed a *Bifidobacterium*-specific primer pair which targets a hypervariable sequence region within the *groEL* gene that is suitable for precise taxonomic identification and detection of all recognized species of the genus *Bifidobacterium* so far. The results showed that the novel designed primer set can specifically differentiate *Bifidobacterium* species from non-bifidobacteria, and as low as 10^4^ cells of *Bifidobacterium* species can be detected using the novel designed primer set on the basis of Illumina Miseq high-throughput sequencing. We also developed a novel protocol to assess the diversity of *Bifidobacterium* species in both human and rat feces through high-throughput sequencing technologies using *groEL* gene as a discriminative marker.

## 1. Introduction

Members of *Bifidobacterium* species, characterized by high G + C Gram-positive, non-motile, non-gas-producing, non-sporulating, anaerobic bacteria, constitute a group of the commensal bacterium of human and animal intestinal microbiota. The contribution of bifidobacteria in maintaining or improving human and animal health has been accepted and some members of the *Bifidobacterium* species have been added as probiotics to various foods [1,2]. Therefore, it is vital to detect and identify bifidobacterial strains precisely and to assess their diversity and population size in the gastrointestinal tract. Within this context, assessment of the *Bifidobacterium* species in complex samples such as feces from humans and animals has attracted great interest from researchers.

With the advent of massive parallel sequencing technologies, the cultivation-independent methods based on the 16S rRNA gene with the help of next-generation sequencing technologies have been extensively recognized as a useful tool for the assessment of *Bifidobacterium* species [3,4,5,6,7]. Among the next-generation sequencing technologies, the MiSeq Illumina sequencing platform had the lowest error rates and highest throughput per run as compared to the 454 GS Junior and Ion Torrent PGM [8]. However, the resolvability of the 16S rRNA gene sequences among closely related bacterial strains is limited. In general, bacterial strains showing more than 97% 16S rRNA gene sequence similarity are usually considered the same species. Because *Bifidobacterium* species reveal a relatively high 16S rRNA gene sequence similarity, markers with higher discriminating power are necessary. The internal transcribed spacer (ITS) region provides a high resolution and can be used to assess population biodiversity in bacterial communities [9]. However, the ITS marker for studying the environmental samples is problematic in that the presence of operon copy number heterogeneity within a genome and the possibility of intragenomic variation in ITS sequence and length may lead to skewed estimates of bacterial communities [10,11].

Alternative target genes, such as *atpD* [12], *recA* [13], *tuf* [14], *dnaK* [15], *tal* [16], *xfp* [17], *rpoC* [18], and *groEL* [19,20,21,22] have been used for the differentiation and identification of *Bifidobacterium* species. These gene markers have been demonstrated to have similar or even higher resolution ability for *Bifidobacterium* species than the 16S rRNA gene. Compared to other molecular markers, the *groEL* gene has more advantages. The *groEL* gene is a ubiquitous housekeeping gene in the genus *Bifidobacterium*, that encodes the heat shock proteins (Hsp60, also known as Cpn60 or GroEL), which play an important role in response to cellular stress. Additional sequences of bifidobacterial strains are available in the Chaperonin Sequence Database [23]. Many experiments have proved that *Bifidobacterium* species have just one copy of the *groEL* gene [24,25,26], which facilitates quantitative analyses of *Bifidobacterium* species. Furthermore, the *groEL* gene sequence identities between *Bifidobacterium* species were much lower than those of 16S rDNA, thus possessing higher resolution power for *Bifidobacterium* species than the 16S rRNA gene.

In this study, a novel protocol was described to assess *Bifidobacterium* species through high-throughput sequencing technologies using *groEL* as a discriminative marker. To test the robustness of the novel designed primer set, we analyzed *Bifidobacterium* species in human and rat fecal samples using the designed primer set on the basis of the MiSeq Illumina sequencing platform.

## 2. Materials and Methods

### 2.1. Bacterial Strains, Culture Media and DNA Extraction

The bifidobacterial strains used in the study were as follows: *B. adolescentis* CCFM626, *B. animalis* subsp. *animalis* CCFM624, *B. animalis* subsp. *lactis* BB12, *B. bifidum* CCFM641, *B. breve* CCFM623, *B. dentium* FJSNT63M4, *B. longum* subsp. *infantis* CCFM666, *B. longum* subsp. *longum* CCFM642, *B. pseudocatenulatum* CCFM749, and *B. pseudolongum* FJSWX2M9. All the bifidobacterial strains were grown in de Man, Rogosa and Sharp (MRS) broth with addition of 0.05% of l-cysteine hydrochloride monohydrate at 37 °C. Seven non-bifidobacterial strains were also used in this study: *Actinomyces odontolyticus* HNSQ3B4, *Bacteroides uniformis* CCFM792, *Escherichia coli* CCFM21, *Enterococcus faecalis* CCFM596, *Lactobacillus acidophilus* CCFM137, *L. plantarum* ST-III (CGMCC no. 0847) and *Rothia dentocariosa* JSWX1B7. All the *Lactobacillus* strains were cultured in MRS broth at 37 °C. *E. coli* was cultured at 37 °C in Luria-Bertani medium. *A. odontolyticus, B. uniformis*, *E. faecalis* and *R. dentocariosa* were grown in Brain Heart Infusion Broth at 37 °C. All the bacteria used in this study came from the Culture Collection of Food Microorganisms of Jiangnan University (Wuxi, China).

Genomic DNA of these bacteria was extracted using the method described previously and subjected to further phenol/chloroform purification using an established protocol [27,28].

### 2.2. Fecal Sample Collection and Genomic DNA Extraction

Feces from humans and from rats were collected in this study. All stool samples from adult humans were collected in sterile containers within 20 min after defecation, transported to the laboratory on ice, and stored at –80 °C until genomic DNA was extracted. Fresh feces from rats were collected in individual sterile EP tubes on ice, which were taken to the laboratory within 2 h and stored at –80 °C for further observation. Fecal samples were homogenized and then subjected to bacterial genome DNA extraction using FastDNA SPIN Kit for Feces (MP Biomedicals; Carlsbad, CA, USA) as per the manufacturer’s protocols. The protocols of the study were approved by the Ethical Committee of Jiangnan University (Wuxi, China).

### 2.3. Phylogenetic Analysis

The phylogenetic relationships among the genus *Bifidobacterium* were constructed using *groEL* gene. The best fitted substitution model for each partition was estimated using Akaike information criterion (AIC) implemented in jModeltest [29]. The model of TIM1 + I + G was chosen for Maximum likelihood (ML) analyses, which were performed with RAxML BlackBox web servers [30].

### 2.4. Bifidobacterium groEL-Specific Primer Design

All available bifidobacterial and some actinobacterial *groEL* gene sequences were retrieved from the GenBank and European Molecular Biology Laboratory (EMBL) nucleotide sequence databases and aligned using the ClustalW software program [31]. To identify *Bifidobacterium* species, a region of 487 or 496 base pairs (bp) located at positions 1066 to 1552 (*B. animalis*) or 1561 (*B. breve*) of the complete *groEL* gene of ca. 1600 bp was chosen as discriminative target sites. Degenerate primers Bif-*groEL*-F (5-TCC GAT TAC GAY CGY GAG AAG CT-3)/Bif-*groEL*-R (5-CSG CYT CGG TSG TCA GGA ACA G-3) for the genus *Bifidobacterium* were manually designed according to multiple sequence alignment. Specificity testing was carried out using PRIMER-BLAST which allows in silico PCR amplification using the National Center for Biotechnology Information (NCBI) nonredundant database as a template [32]. An additional specificity test was conducted by PCR amplification using genomic DNA extracted from known bacterial species: *A. odontolyticus, B. uniformis*, *E. coli*, *E. faecalis*, *L. acidophilus*, *L. plantarum* and *R. dentocariosa*. The primers were synthesized by the Shanghai Sangon Biological Science & Technology Company (Shanghai, China) and used for the PCR amplification.

### 2.5. PCR Amplification, Quantification, and Sequencing

The selected partial *groEL* gene sequences from microbial genome DNA were PCR amplified using the barcoded fusion primers Bif-*groEL*-F/Bif-*groEL*-R designed during this study. PCR amplifications were performed using a 50 μL total volume consisting of 1 μL of the target DNA, 25 μL Premix Taq (TaKaRa, Dalian, China), 1 μL of each primer (20 μM) and 22 μL of double distilled water (ddH_2_O). The PCR amplification procedures were pre-denaturation at 95 °C for 4 min, followed by 30 cycles consisting of denaturation at 95 °C for 30 s, annealing for 30 s at 60 °C, extension for 50 s at 72 °C and the final step at 72 °C for 10 min. In addition, The V3–V4 hypervariable sequence region of the 16S rRNA gene was amplified with the forward primer (341F: 5-CCT AYG GGR BGC ASC AG-3) and reverse primer (806R: 5-GGA CTA CNN GGG TAT CTA AT-3) according to the previous protocols [33].

All the PCR amplification products obtained following amplification of the *groEL* and 16S rRNA gene sequences were excised from the agarose gel, purified using a QIAquick Gel Extraction Kit (Qiagen, Hilden, Germany) and quantified by Quant-iT PicoGreen dsDNA Assay Kit (Life Technologies, Carlsbad, CA, USA) following the manufacturer’s recommendations. DNA amplicon libraries were prepared using TruSeq DNA LT Sample Preparation Kit (Illumina, San Diego, CA, USA) and sequenced on the MiSeq Illumina platform using the MiSeq v3 Reagent Kit (600 cycles) following instructions provided by the manufacturer.

### 2.6. Evaluation of the Sensitivity of the Novel Designed Primer Set

The detection sensitivity and accuracy of the primer set Bif-*groEL*-F/Bif-*groEL*-R were evaluated employing known DNA amounts, ranging from 0.01 to 40 ng, of the artificial sample from 10 different bifidobacterial taxa. The *groEL* gene copy numbers were estimated using “Calculator for Determining the Number of Copies of a Template” (URI Genomics & Sequencing Center) [34]. Thus, colony-forming units (CFU) could be calculated on the basis of the *groEL* gene copy numbers predicted above.

### 2.7. Bioinformatic Sequence Analysis

Sequence reads were processed with the QIIME package version 1.9.1 (Quantitative Insights Into Microbial Ecology, Flagstaff, AZ, USA) [35]. The raw sequences with a lower quality score and short-read length compared to target sequences were first removed. Sequences were also removed if they contained ambiguous bases or mismatches in primers. Only pair-end reads overlapping longer than 10 bp and without any mismatch were assembled following their overlap sequences, and the unassembled reads were discarded. Barcode and sequencing primers from the above assembled sequences were trimmed.

Each sample’s high-quality reads were clustered into operational taxonomic units (OTUs) for further taxonomic analyses. Taxonomic identification of OTUs for the *groEL* sequences was performed through comparison to the Chaperonin Sequence Database [23]. The OTUs of V3–V4 region sequences were assigned to a taxonomy with the naive Bayes classifier of the Ribosomal Database Project (RDP) [36], and all OTUs with representative sequences from each cluster were combined and aligned against the Greengenes core set in QIIME with the PyNAST aligner [35,37]. Similarities among the microbial communities were estimated using cluster heatmap analysis with the R software for statistical computing [38].

### 2.8. Real-Time qPCR

The total and main bifidobacterial numbers of human feces were determined by SYBR Green-based qPCR using a CFX96 real-time PCR detection system (Bio-Rad Laboratories, Hercules, CA, USA) as previously described with some modifications [22,39]. The total bifidobacteria and the main bifidobacteria of *B. pseudocatenulatum* and *B. longum* subsp. *longum* from human feces were quantified through qPCR amplifications using the primers previously described in 20 μL volume using 96-well plates in triplicate [22]. For quantification of the genus *Bifidobacterium*, *B. longum* subsp. *longum* CCFM642 was used as the standard strain. *B. pseudocatenulatum* CCFM749 and *B. longum* subsp. *longum* CCFM642 were also used as qPCR controls for species-specific quantification. The target bifidobacterial population was expressed as Log_10_ bifidobacteria per gram of wet stool.

### 2.9 Statistical Analysis

All data were expressed as means ± standard deviation (SD). The statistical analyses of differences between two groups were analyzed using Student’s *t*-test. The analyses were carried out with SPSS version 16.0 (SPSS Inc., Chicago, IL, USA), and statistical significance was accepted at least at the 5% level.

## 3. Results

### 3.1. Comparative Analysis of the groEL and 16S rRNA Gene

**Figure 1:**
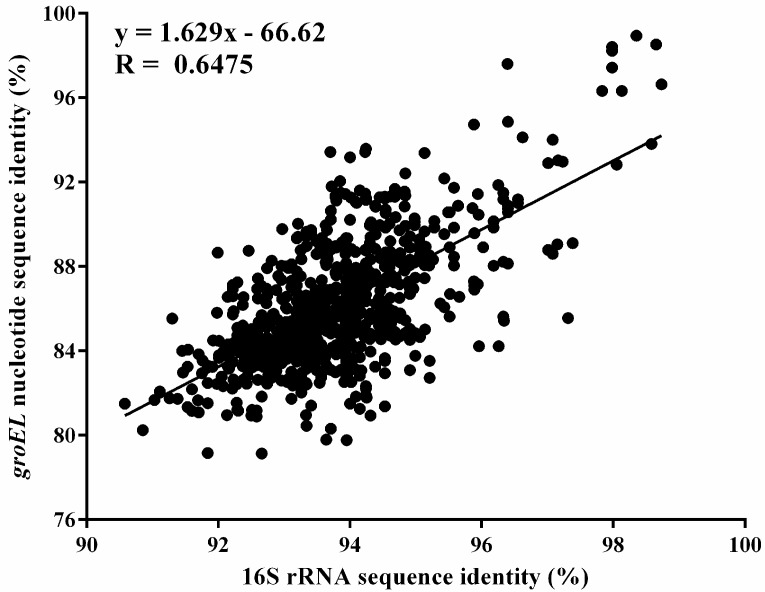
Association between the degree of sequence identity of 16S rDNA and the *groEL* gene for pairs of genomes assigned to the same species.

The *groEL* and 16S rRNA gene nucleotide sequences of *Bifidobacterium* species (download from NCBI and EMBL databases) were compared by BLAST [40] (Table 1). When comparing the two sets of sequences, we found that the lowest value of the pairwise similarities of the *groEL* gene is 79.1% (*B. magnum* and *B. tsurumiense*) and the average pairwise similarities value is 86.3% whereas the lowest value of the pairwise similarities of the 16S rRNA gene is 90.9% (*B. magnum* and *B. crudilactis*) and the average pairwise similarities value is 93.8%. Thus, the *groEL* gene provids higher resolution power than that provided by the 16S rRNA gene. We also plotted the average pairwise similarities values targeted for the *groEL* gene against the average pairwise similarities values corresponding to 16S rRNA gene sequences for classification of microorganisms at the same taxonomic rank. Using the regression shown in Figure 1, we found that the variation trend of the average pairwise similarities values were similar between the *groEL* and 16S rRNA genes.

### 3.2. Phylogenetic Analysis of the Partial groEL Gene

**Figure 2:**
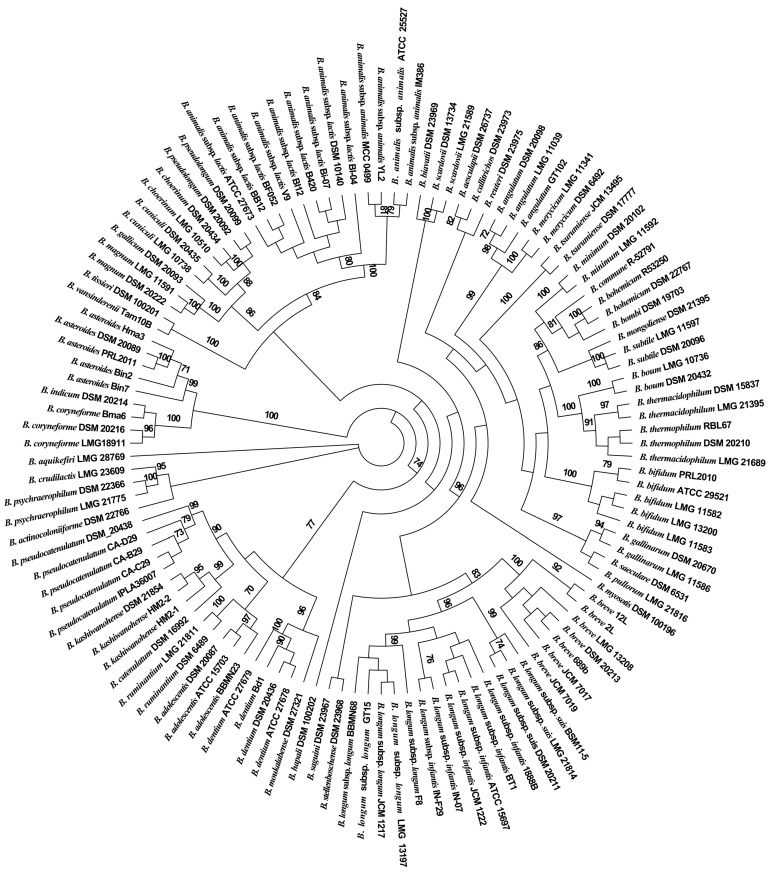
A maximum likelihood phylogeny of the selected partial *groEL* gene sequences for the genus *Bifidobacterium*. Bootstrap values above 70% are given on the branches based on 1000 replicates of the phylogenetic tree.

The study determined the selected partial sequences of the *groEL* gene and V3–V4 region of the 16S rRNA gene of 120 bifidobacterial strains from 54 species (subspecies) (Table S1). The results by BLAST revealed that the lowest value of the pairwise similarities of the partial *groEL* gene is 74.9% (*B. commune* R-52791 and *B. animalis* subsp. *lactis* BB12) and the average pairwise similarities value is 84.9%. We used the MEGA software (Version 5.1) to align the selected partial *groEL* gene and V3–V4 region sequences determined in this study [41]. As shown in Figure 2, a ML analysis of *groEL* gene sequences from the bifidobacterial strains conducted in the phylogenetic dendrogram revealed that the *Bifidobacterium* species were grouped into six clusters. Moreover, the closely related *Bifidobacterium* species (i.e., subspecies) fell into the same clusters, whereas different *Bifidobacterium* species were categorized into different clusters. Specifically, the closely related taxa such as members of *B. longum* subsp. *longum*, *B. longum* subsp. *infantis* and *B. longum* subsp. *suis*, as well as *B. animalis* subsp. *animalis* and *B. animalis* subsp. *lactis* can be distinguished on the basis of the selected partial *groEL* gene, confirming that the partial *groEL* gene possessed the high taxonomic and phylogenetic resolution for identification and differentiation of the *Bifidobacterium* species. However, as shown in Figure S1, the neighbor-joining tree on the basis of the V3–V4 region sequences of the 16S rRNA gene showed that some of the same *Bifidobacterium* species were grouped into different clusters, demonstrating that the V3–V4 region sequences of the 16S rRNA gene lacked sufficient resolution for distinguishing different *Bifidobacterium* species.

### 3.3. Specificity, Accuracy and Sensitivity of the Novel Designed Primer Set

**Figure 3:**
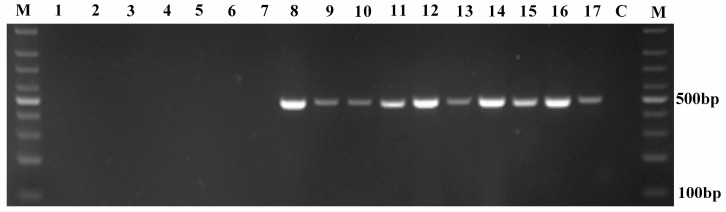
Specificity of PCR amplification of the selected partial *groEL* gene using the novel designed primer set. M, marker; 1, *A. odontolyticus*, 2, *B. uniformis*, 3, *E. coli*, 4, *E. faecalis*, 5, *L. acidophilus*, 6, *L. plantarum*; 7, *R. dentocariosa*, 8, *B. adolescentis*; 9, *B. animalis* subsp. *animalis*; 10, *B. animalis* subsp. *lactis*; 11, *B. bifidum*; 12, *B. breve*; 13, *B. dentium*; 14, *B. longum* subsp. *infantis*; 15, *B. longum* subsp. *longum*; 16, *B. pseudocatenulatum*; 17, *B. pseudolongum*; C, control.

*In silico* PCR through PRIMER-BLAST generates only an amplicon for bifidobacterial genomes, suggesting the bifidobacterial specificity of the primer set. We also performed PCR amplification using genomic DNA extracted from known bacterial species including ten bifidobacterial strains and seven non-bifidobacterial strains. As shown in Figure 3, the results revealed that a PCR amplification product was obtained only when template DNA was extracted from *Bifidobacterium* species, whereas no PCR amplification product was observed when DNA genome from any of the other investigated non-bifidobacterial strains was used as a template. Therefore, the novel designed primer set can specifically differentiate *Bifidobacterium* species from other bacterial species tested in this study.

**Figure 4:**
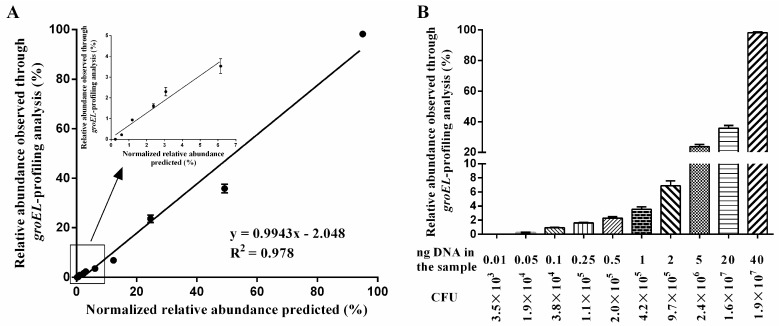
Accuracy and the limit of detection of the novel designed primer set. (**A**) Relationship between normalized relative abundance predicted of *Bifidobacterium* species and relative abundance observed through *groEL*-profiling analysis. (**B**) The limit of detection (LOD) of the novel designed primer set based on the selected partial *groEL* gene. CFU: Colony-forming units.

To evaluate the accuracy and sensitivity of the novel designed primer set, we developed artificial samples consisting of known DNA amounts of different bifidobacterial species. The genomic DNA from these *Bifidobacterium* species served as a template for PCR amplification with the novel designed primer set, and the amplicons were sequenced on the MiSeq Illumina sequencing platform. Figure 4A shows strong correlation of the relative abundances of taxa through comparison between known bifidobacterial composition of the artificial samples and retrieved results through *groEL*-profiling analysis. Specifically, the minimum DNA amount of detectable bifidobacterial species was 0.05 ng, which corresponds to *Bifidobacterium* species of 10^4^ CFU at concentration. Therefore, the limit of detection (LOD) of the novel designed primer set based on the MiSeq Illumina sequencing platform was 10^4^ CFU/mL (Figure 4B).

### 3.4. Comparison of Resolving Power between the Partial groEL Gene and V3–V4 Region of 16S rRNA

To evaluate the efficacy of the *groEL*-based primer set designed in the study, we sequenced the partial *groEL* gene and V3–V4 region of 16S rRNA amplicons obtained by PCR amplification using the same genomic DNA from human and rat fecal samples. As shown in Table 2, MiSeq sequencing analysis of feces samples from eight humans generated 136,488 and 181,257 high-quality and classifiable sequences corresponding to the *groEL* gene and 16S rRNA gene, respectively, and average sequence reads of the two genes were 17,061 and 22,657 per sample. For eight rat fecal samples, 150,875 and 152,033 high-quality and classifiable sequences were obtained for the *groEL* gene and 16S rRNA gene, respectively, and average sequence reads of the two genes were 18,859 and 19,004 per sample.

**Figure 5:**
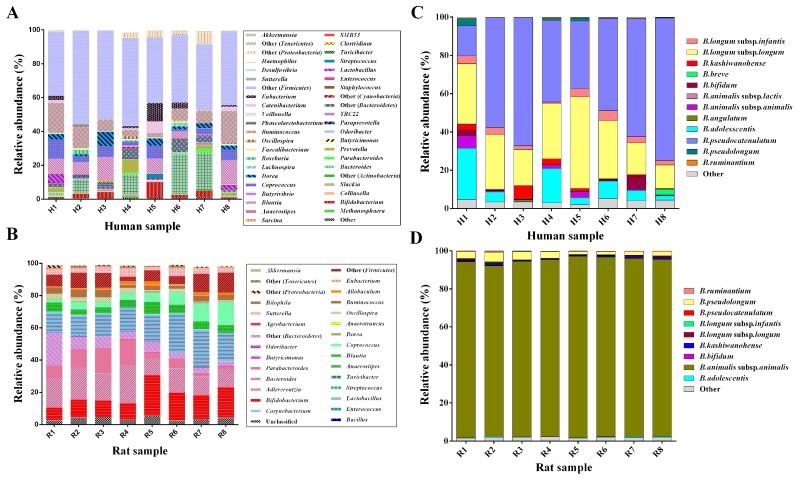
16S rRNA gene–based and *groEL* gene-based profiling of human and rat fecal samples involving Bif-*groEL*-F/Bif-*groEL*-R and 341F/806R primer pairs. (**A**) Bar plots of the microbial composition at the genus level of the eight analyzed human samples and (**B**) of the eight analyzed rat samples. (**C**) Bar plots of the microbial composition at the species level of the eight analyzed human samples and (**D**) of the eight analyzed rat samples.

To evaluate the robustness of the designed primer pair in determining the bifidobacterial community composition in complex ecosystems, the bifidobacterial profiles identified were compared using the new designed primer pair Bif-*groEL*-F/Bif-*groEL*-R and the bifidobacterial profiles obtained with the primer set 341F/806R as described previously in each case using the same genomic DNA. As shown in Figure 5A,B, when assessing the diversity of bifidobacteria, using the universal primer set corresponding to the V3–V4 region of the 16S rRNA gene, on average, only about 2.8% and 14.4% of the tens of thousands of reads generated for the human and rat fecal samples were assigned to the genus *Bifidobacterium*. When using the primer pairs Bif-*groEL*-F/Bif-*groEL*-R, we amplified a region of the *groEL* gene from *Bifidobacterium* species, and the results revealed that almost all the sequences could be assigned to the genus *Bifidobacterium* (Figure 5C,D). Furthermore, the primer pairs targeted to the partial *groEL* gene could identify bifidobacteria at the species level, in contrast to the universal primer set of the V3–V4 region of 16S rDNA at the genus level.

### 3.5. Comparison of Bifidobacterium Species between Humans and Rats

**Figure 6:**
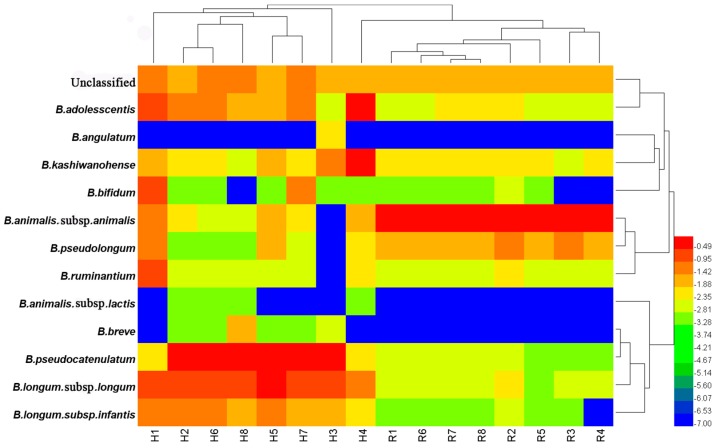
Relative abundance of *Bifidobacterium* species was visualized using a heatmap determined using the sequence data obtained from human and rat fecal samples, with a high percentage of species belonging to the genus *Bifidobacterium* indicated in red and low percentages in blue. Each row on the *y*-axis represents a *Bifidobacterium* species, and each column on the *x*-axis represents a sample.

**Figure 7:**
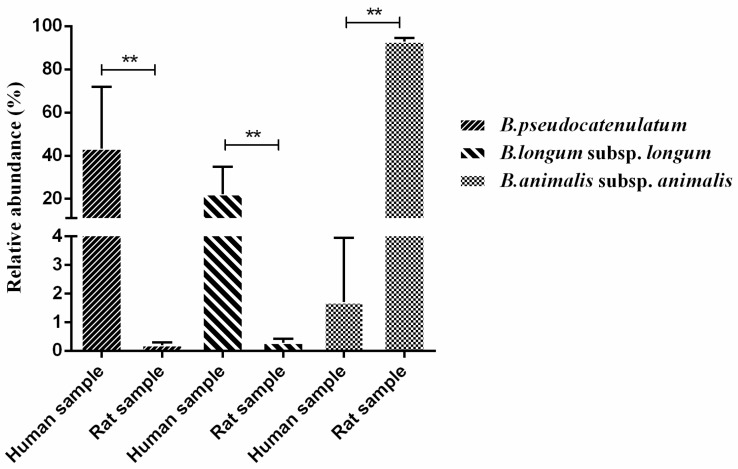
Differences in relative abundance of selected *Bifidobacterium* species. * *p* < 0.05; ** *p* < 0.01 according to one-way analysis of variance and Duncan’s multiple comparisons test.

As shown in Figure 5C,D, 12 *Bifidobacterium* species are contained in human fecal samples, and nine are contained in rat fecal samples. Notably, the predominant *Bifidobacterium* species for human gut bacteria were *B. pseudocatenulatum* and *B. longum* subsp. *longum*. The dominate gut bifidobacteria of rats were *B. animalis* subsp. *animalis*. Furthermore, when cluster heatmap analysis (Figure 6) was performed to visualize the differences in the composition of *Bifidobacterium* species from human and rat fecal samples, these *Bifidobacterium* species from humans and rats formed two distinct blocks on the heatmap. Notably, the relative abundances of *B. pseudocatenulatum* and *B. longum* subsp. *longum* (humans) and *B. animalis* subsp. *animalis* (rats) are significantly different among gut bifidobacteria from humans and rats (*p* < 0.05) (Figure 7).

### 3.6. qPCR-Based Determination of Main Bifidobacterial Numbers

To further quantify the main bifidobacterial numbers in human feces, qPCR analysis was carried out in a CFX96 real-time PCR detection system. As shown in Table S2, the results of the main bifidobacterial community composition in human feces from qPCR were in accord with those from the new method of using the designed primer set on the basis of the MiSeq Illumina sequencing platform.

## 4. Discussion

To identify *Bifidobacterium* species on the basis of the MiSeq Illumina sequencing platform, the following criteria should be met: (1) The target gene must be ubiquitous in the genus *Bifidobacterium*; (2) the target gene must have high resolution power; (3) the target gene used for primer binding must include a sequence containing a hypervariable region flanked by two constant regions; (4) the PCR amplification region in the target gene must comprise no more than 500-bp nucleotide sequences; and (5) many sequences of the target gene must be available.

The *groEL* gene, a single-copy housekeeping gene, is ubiquitous in the genus *Bifidobacterium*. According to the multiple sequence alignment method used in the study, we selected a fragment of about 490 bp for PCR amplification with the designed degenerate primers, and the selected partial *groEL* nucleotide sequence identities among different *Bifidobacterium* species ranged from 74.3 to 96.7% (mean 85.0%). In addition, the phylogenetic tree depicted using the selected region of the *groEL* gene or even complete *groEL* gene nucleotide sequences (about 1600 bp) of *Bifidobacterium* species [18,20,21] was similar to the one based on the 16S rRNA gene sequences [3,42]. A significant correlation existed between the genetic distances of the *groEL* gene nucleotide sequences and those of the 16S rDNA nucleotide sequences [21]. Furthermore, the 16S rDNA nucleotide sequence identities among all the *Bifidobacterium* species ranged from 90.6 to 99.9% [42]. Notably, the resolving ability of the 16S rRNA gene is limited among some closely related *Bifidobacterium* species. For example, *B. catenulatum* and *B. pseudocatenulatum* cannot be distinguished because they share 98.5% nucleotide sequence similarity in their 16S rRNA genes sequences [18]. However, the phylogenetic dendrogram delineated in this study showed that *B. catenulatum* and *B. pseudocatenulatum* could be easily distinguished because of the 93.9% nucleotide sequence similarity between the two organisms in the selected region of the *groEL* gene. Moreover, at the subspecies level, *B. animalis* subsp. *animalis* and *B. animalis* subsp. *lactis*, as well as *B. longum* subsp. *infantis*, *B. longum* subsp. *longum* and *B. longum* subsp. *suis*, share 16S rRNA nucleotide sequence similarities are all above 99% [43]. The selected region of the *groEL* gene nucleotide sequence identities between *B. animalis* subsp. *animalis* and *B. animalis* subsp. *lactis*, as well as *B. longum* subsp. *infantis* and *B. longum* subsp. *longum* were 94.1% and 98.2%, respectively. In contrast, the selected region of the *groEL* gene used during the study displayed a considerably higher resolving ability between these closely related species in *Bifidobacterium* species than did the 16S rRNA gene. What is more, the reference databases (NCBI, EMBL and Chaperonin Sequence Database) provide ample nucleotide sequences of the *groEL* gene for further sequence alignment. Overall, the selected region of the *groEL* gene fulfils all of the prerequisites to serve as a reliable alternative target marker gene for distinguishing different *Bifidobacterium* species based on the MiSeq Illumina sequencing platform.

To characterize the specificity and LOD of the developed method using the novel designed primer set on the high-throughput sequencing platform, we prepared samples spiked with the known concentrations of *Bifidobacterium* species and other bacteria for sequencing based on the MiSeq Illumina platform. The results demonstrated that the developed method could discriminate different *Bifidobacterium* species, and concentrations of *Bifidobacterium* species as low as 10^4^ CFU/mL could be detected.

To prove the robustness of the novel designed primer set, two kinds of different fecal samples derived from humans and rats were selected for further analysis. Based on the MiSeq Illumina sequencing platform, we identified the *Bifidobacterium* species of both sets of fecal samples. The results showed that the diversity of bifidobacterial composition in human samples is greater than that in rat samples. One possible reason may be that diets varied among different people, whereas diets in different rats did not vary.

It is known that there may be drawbacks to each method. Maybe there is no one gene that can differentiate all *Bifidobacterium* species. One possible limitation of *groEL* gene is that a high similarity of *groEL* gene similarity values between different bifidobacterial species is present. For example, there are high similarity values between *B. thermophilum* and *B. thermacidophilum* subsp. *thermacidophilum*, *B. longum* subsp. *infantis* and *B. longum* subsp. *longum*. Consequently, we should be careful when differentiating these different *Bifidobacterium* species using *groEL* gene. Maybe *groEL* gene combined with more genes like 16S rRNA, *rpoB* and *clpC* are good in differentiating these different bifidobacterial species with a high similarity of *groEL* similarity values [44].

## 5. Conclusions

In conclusion, a *Bifidobacterium*-specific primer pair that targets a hypervariable region of about 490 bp within the *groEL* gene can specifically differentiate *Bifidobacterium* species from bacterial species, and the LOD of the novel designed primer set based on the MiSeq Illumina sequencing platform was 10^4^ CFU/mL. In addition, the novel designed primer pair can identify bifidobacteria at the species level from human and rat fecal samples on the basis of the MiSeq Illumina sequencing platform. The results demonstrated that the predominant *Bifidobacterium* species for human gut bacteria were *B. pseudocatenulatum* and *B. longum* subsp. *longum*, and the predominant gut bifidobacteria of rats were *B. animalis* subsp. *animalis*.

## Figures and Tables

**Table 1:** List of *Bifidobacterium* strains used for comparative analysis.

Number	*Bifidobacterium* Species	Strain ^a^	GenBank Accession no. of *groEL* Gene Sequences	GenBank Accession no. of 16S rRNA Gene Sequences
1	*B. adolescentis*	ATCC 15703	AP009256	NR_074802
2	*B. angulatum*	JCM 7096	AP012322	LC071846
3	*B. animalis* subsp. *animalis*	ATCC 25527 = LMG 10508	CP002567	JGYM01000004
4	*B. animalis* subsp. *lactis*	BB12	CP001853	GU116483
5	*B. asteroides*	Bin2	NZ_KQ033859	EF187231
6	*B. biavatii*	DSM 23969	JGYN01000004	JGYN01000007
7	*B. bifidum*	PRL2010	CP001840	CP001840
8	*B. bohemicum*	R53250	FMAM01000001	FMAM01000014
9	*B. boum*	LMG 10736 = JCM 1211	JGYQ01000016	LC071799
10	*B. breve*	JCM 1192	AP012324	LC071793
11	*B. callitrichos*	DSM 23973	JGYS01000001	JGYS01000004
12	*B. catenulatum*	DSM 16992	AP012325	NR_041875
13	*B. choerinum*	ATCC 27686 = LMG 10510	JGYU01000001	D86186
14	*B. coryneforme*	Bma6	KQ033865	EF187237
15	*B. crudilactis*	LMG 23609	NZ_JHAL01000002	NZ_JHAL01000001
16	*B. cuniculi*	LMG 10738	JGYV01000008	JX986964
17	*B. dentium*	JCM 1195	AP012326	LC071795
18	*B. gallicum*	DSM 20093 = LMG 11596	NZ_ABXB03000002	ABXB03000004
19	*B. gallinarum*	DSM 20670 = JCM 6291	NZ_JDUN01000004	D86191
20	*B. indicum*	LMG 11587 = DSM 20214 = JCM1302	CP006018	D86188
21	*B. magnum*	DSM 20222 = JCM 1218	NZ_ATVE01000001	D86193
22	*B. merycicum*	DSM 6492 = JCM 8219	NZ_JDTL01000006	D86192
23	*B. minimum*	DSM 20102 = ATCC 27538	NZ_ATXM01000001	M58741
24	*B. kashiwanohense*	HM2-1	AB578933	AB491757
25	*B. longum* subsp. *infantis*	ATCC 15697	CP001095	NR_043437
26	*B. longum* subsp. *longum*	BBMN68	CP002286	GQ380695.1
27	*B. mongoliense*	DSM 21395	JGZE01000001	AB433856
28	*B. pseudocatenulatum*	JCM 1200	AP012330	LC071796
29	*B. pseudolongum*	PV8-2	CP007457	CP007457
30	*B. pullorum*	DSM 20433 = JCM 1214	NZ_JDUI01000001	D86196
31	*B. reuteri*	DSM 23975	NZ_JDUW01000002	NZ_JDUW01000049
32	*B. ruminantium*	DSM 6489 = JCM 8222	NZ_JHWQ01000003	D86197
33	*B. saeculare*	DSM 6531	JGZM01000001	D89328
34	*B. scardovii*	JCM 12489	AP012331	AP012331
35	*B. stellenboschense*	DSM 23968	NZ_JGZP01000019	JGZP01000012
36	*B. stercoris*	JCM 15918	JGZQ01000008	NZ_JDUX01000017
37	*B. subtile*	DSM 20096	NZ_AUFH01000005	D89378
38	*B. thermacidophilum* subsp. *porcinum*	LMG 21689	JGZS01000003	NZ_JGZS01000003
39	*B. thermophilum*	RBL67	CP004346	DQ340557
40	*B. tsurumiense*	DSM 17777 = OMB115	NZ_AUCL01000007	AB241106

^a^ ATCC, American Type Culture Collection; DSM, Deutsche Sammlung von Mikroorganismen und Zellkulturen; JCM, Japanese Collection of Microorganisms; LMG, Laboratorium voor Microbiologie, University of Ghent.

**Table 2:** Overview of sequencing results for each sample.

Sample ID	Sequence Number ^a^ (16S)	OTU Number ^b^ (16S)	Sequence Number (*groEL*)	OTU Number (*groEL*)
H1	8774	3187	8583	2044
H2	29297	1566	20519	3050
H3	26457	1511	23778	2755
H4	28524	2632	18206	3176
H5	18197	970	15605	2536
H6	19625	2045	15297	3943
H7	13352	1137	14950	3457
H8	37031	3429	19550	4349
R1	13986	1351	9341	736
R2	9721	1279	9385	815
R3	13187	1462	34297	1665
R4	16230	1404	27903	1545
R5	43429	936	17599	967
R6	19969	596	19369	1060
R7	18249	723	19835	1111
R8	17262	930	13146	870

^a^ The sequence number refers to the count of assembled sequences after quality filtering. ^b^ The OTU (Operational Taxonomic Units) number is presented for all sequences without rarefaction.

## Supplementary Materials

The following are available online at www.mdpi.com/2073-4425/8/11/336/s1. Figure S1: Phylogenetic tree based on the V3–V4 region sequences of the 16S rRNA gene. The tree was constructed by the neighbor-joining distance method with bootstrap values calculated from 1000 trees, Table S1: List of *Bifidobacterium* strains used for phylogenetic analysis of the selected partial *groEL* gene and the V3–V4 region of the 16S rRNA gene.
