# Supplementary material for: Assessment of Bifidobacterium Species Using groEL Gene on the Basis of Illumina MiSeq High-Throughput Sequencing
Source: Genes (Basel). 2017 Nov 21;8(11):336. doi: 10.3390/genes8110336 (PMC5704249; doi:10.3390/genes8110336)
Supplement: Supplementary file 1 [file genes-08-00336-s001.pdf]

Supplementary Materials: Assessment of *Bifidobacterium* Species Using *groEL* Gene on the Basis of Illumina MiSeq High-Throughput Sequencing

Lujun Hu, Wenwei Lu, Linlin Wang, Mingluo Pan, Hao Zhang, Jianxin Zhao and Wei Chen

Table S1. List of *Bifidobacterium* strains used for phylogenetic analysis of the selected partial *groEL* gene and the V3–V4 region of the 16S rRNA gene.

Number	*Bifidobacterium* species	strain	GenBank accession no.of *groEL*	Strain	GenBank accession no. of 16S rRNA
1	*B. actinocoloniiforme*	DSM 22766	NZ_CP011786	DSM 22766	FJ858731
2	*B. adolescentis*	ATCC 15703	AP009256	ATCC 15703	NR_074802
3	*B. adolescentis*	DSM 20087	NZ_JNKM01000002	DSM 20087	NZ_JNKM01000001
4	*B. adolescentis*	BBMN23	CP010437	BBMN23	GQ380694
5	*B. aesculapii*	DSM 26737	NZ_BCFK01000003	MRM 3/1	KC807989
6	*B. angulatum*	LMG 11039	JGYL01000001	B677	NR_036853
7	*B. angulatum*	JCM 7096 = DSM 20098 = ATCC 27535	AP012322	DSM 20098	LC071846
8	*B. angulatum*	GT102	CP014241	Fn1	AB489093
9	*B. animalis* subsp. *animalis*	ATCC 25527 = LMG 10508	CP002567	ATCC 25527	X70971
10	*B. animalis* subsp. *animalis*	YL2	CP015407	YL2	KR364745
11	*B. animalis* subsp. *animalis*	MCC 0499	NZ_AWFN01000003	MCC 0499	AWFN01000009
12	*B. animalis* subsp. *animalis*	IM386	NZ_CBUQ010000007	IM386	NZ_CBUQ010000003
13	*B. animalis* subsp. *lactis*	Bb12	CP001853	Bb12	GU116483
14	*B. animalis* subsp. *lactis*	ATCC 27673	CP003941	ATCC 27673	NZ_AWFP01000017
15	*B. animalis* subsp. *lactis*	B420	CP003497	BL2	AY700230
16	*B. animalis* subsp. *lactis*	BF052	CP009045	CECT 8145	CBWX010000074
17	*B. animalis* subsp. *lactis*	Bi-07	CP003498	IDCC 4301	EF589111
18	*B. animalis* subsp. *lactis*	Bl12	CP004053	LCR22	HQ259732
19	*B. animalis* subsp. *lactis*	DSM 10140	CP001606	LCR26	HQ259736
20	*B. animalis* subsp. *lactis*	V9	CP001892	P2N1	KU821112
21	*B. animalis* subsp. *lactis*	Bl-04	CP001515	PL3	DQ360843
22	*B. asteroides*	Bin2	NZ_KQ033859	Bin2	EF187231
23	*B. asteroides*	DSM 20089	NZ_JDTU01000022	ATCC 29510	M58730
24	*B. asteroides*	PRL2011	CP003325	PRL2011	CP003325
25	*B. asteroides*	Bin7	NZ_KQ033885	Bin7	EF187234
26	*B. asteroides*	Hma3	NZ_KQ034040	Hma3	EF187236
27	*B. biavatii*	DSM 23969	JGYN01000004	DSM 23969	JGYN01000007
28	*B. bifidum*	ATCC 29521= JCM 1255 = DSM 20456	AP012323	ATCC 29521	AWSW01000028
29	*B. bifidum*	PRL2010	CP001840	PRL2010	NC_014638
30	*B. bifidum*	LMG 11582	NZ_JSDY01000004	BF2	AY694148
31	*B. bifidum*	LMG 11583	NZ_JSDZ01000011	LMG 11583	JSDZ01000005
32	*B. bifidum*	LMG 13200	NZ_JSEB01000005	KCTC 3202	U25951
33	*B. bohemicum*	DSM 22767	JGYP01000002	DSM 22767	NZ_JDUS01000025
34	*B. bohemicum*	R53250	FMAM01000001	R53250	FMAM01000014

35	*B. boum*	LMG 10736 = JCM 1211	JGYQ01000016	JCM 1211	D86190
36	*B. boum*	DSM 20432	NZ_JHWO01000001	KCTC 3227	GU361814
37	*B. breve*	JCM 1192 = DSM 20213	AP012324	JCM 1192	LC071793
38	*B. breve*	2L	NZ_AWUG01000001	2L	AWUG01000001
39	*B. breve*	12L	CP006711	12L	CP006711
40	*B. breve*	689b	CP006715	689b	CP006715
41	*B. breve*	LMG 13208	NZ_JGYR01000006	LMG 13208	JGYR01000006
42	*B. breve*	JCM 7019	CP006713	JCM 7019	AF491836
43	*B. breve*	JCM 7017	CP006712	JCM 7017	AF491835
44	*B. callitrichos*	DSM 23973	JGYS01000001	DSM 23973	JGYS01000004
45	*B. catenulatum*	DSM 16992	AP012325	DSM 16992	NR_041875
46	*B. choerinum*	ATCC 27686 = LMG 10510	JGYU01000001	ATCC 27686	D86186
47	*B. choerinum*	DSM 20434	NZ_AXVO01000013	JCM 7082	LC269352
48	*B. commune*	LMG 28292 = R-52791	FMBL01000001	LMG 28292	LK054489
49	*B. coryneforme*	DSM 20216	NZ_JDUF01000005	DSM 20216	NZ_JDUF01000029
50	*B. coryneforme*	ATCC 25911 = LMG 18911	CP007287	ATCC 25911	M58733
51	*B. coryneforme*	Bma6	KQ033865	Bma6	EF187237
52	*B. crudilactis*	LMG 23609	NZ_JHAL01000002	LMG 23609	NZ_JHAL01000001
53	*B. cuniculi*	LMG 10738	JGYV01000008	LMG 10738	JX986964
54	*B. cuniculi*	ATCC 27916 = DSM 20435	NZ_JDUL01000086	ATCC 27916	M58734
55	*B. dentium*	DSM 20436 = JCM 1195	AP012326	JCM 1195	LC071795
56	*B. dentium*	Bd1	CP001750	Bd1	CP001750
57	*B. dentium*	ATCC 27678	NZ_ABIX02000002	ATCC 27678	ABIX02000002
58	*B. dentium*	ATCC 27679	NZ_GL405225	ATCC 27679	AEEQ01000018
59	*B. gallicum*	DSM 20093 = LMG 11596 = JCM 8224	NZ_ABXB03000002	JCM 8224	LC071850
60	*B. gallinarum*	DSM 20670 = JCM 6291	NZ_JDUN01000004	JCM 6291	D86191
61	*B. gallinarum*	LMG 11586	JGYX01000003	KCTC 3235	GU361821
62	*B. indicum*	LMG 11587 = DSM 20214 = JCM 1302	CP006018	JCM 1302	D86188
63	*B. kashiwanohense*	DSM 21854 = JCM 15439	AP012327	DSM 21854	AP012327
64	*B. kashiwanohense*	HM2-1	AB578933	HM2-1	AB491757
65	*B. kashiwanohense*	HM2-2	AB491759	HM2-2	AB425276
66	*B. longum* subsp. *infantis*	ATCC 15697	CP001095	ATCC 15697	NR_043437
67	*B. longum* subsp. *infantis*	BT1	CP010411	BT1	AY699578
68	*B. longum* subsp. *infantis*	JCM 1222	AP010889	ATCC 15677	M58738
69	*B. longum* subsp. *infantis*	IN-07	NZ_BCYF01000057	LCR2	HQ259729
70	*B. longum* subsp. *infantis*	IN-F29	NZ_BCYG01000044	YIT 4019	AB924514
71	*B. longum* subsp. *infantis*	1888B	NAQJ01000009	YIT 4081	AB924516
72	*B. longum* subsp. *longum*	F8	FP929034	YIT4021	AB437359
73	*B. longum* subsp. *longum*	JCM 1217	AP010888	KCTC 3128	GU361823
74	*B. longum* subsp. *longum*	BBMN68	CP002286	BBMN68	GQ380695
75	*B. longum* subsp. *longum*	GT15	CP006741	LCR6	HQ259741
76	*B. longum* subsp. *longum*	LMG 13197	NZ_JGYZ01000008	LCR27	HQ259737
77	*B. longum* subsp. *longum suis*	BSM11-5	NZ_MOAE01000002	YIT 4108	AB924531
78	*B. longum* subsp. *longum suis*	ATCC 27533=DSM20211=JCM 1269	NZ_JDUC01000003	ATCC 27533	NR_044693
79	*B. longum* subsp. *longum suis*	LMG 21814	NZ_JGZA01000001	LMG 21814	JGZA01000002
80	*B. magnum*	DSM 20222 = JCM 1218	NZ_ATVE01000001	JCM 1218	D86193

81	*B. magnum*	LMG 11591	JGZB01000003	ATCC 27540	M58740
82	*B. merycicum*	DSM 6492 = JCM 8219	NZ_JDTL01000006	JCM 8219	D86192
83	*B. merycicum*	LMG 11341	JGZC01000010	KCTC 3369	GU361825
84	*B. minimum*	DSM 20102 = ATCC 27538 = JCM 5821	NZ_ATXM01000001	ATCC 27538	M58741
85	*B. minimum*	LMG 11592	JGZD01000009	YIT 4097	AB437350
86	*B. mongoliense*	DSM 21395 = JCM 15461	JGZE01000001	DSM 21395	AB433856
87	*B. moukalabense*	DSM 27321	AZMV01000007	JCM 18751	AB821293
88	*B. pseudocatenulatum*	DSM 20438 = JCM 1200 = LMG 10505	AP012330	JCM 1200	LC071796
89	*B. pseudocatenulatum*	IPLA36007	JEOD01000015	IPLA36007	JEOD01000008
90	*B. pseudocatenulatum*	CA-B29	NZ_BCXZ01000029	CCFM8395	KJ803952
91	*B. pseudocatenulatum*	CA-C29	NZ_BCYA01000046	CCFM8396	KJ803953
92	*B. pseudocatenulatum*	CA-D29	NZ_BCYB01000037	CCFM8398	KJ803955
93	*B. pseudolongum* subsp. *globosum*	DSM 20092	NZ_JHWN01000002	JCM 5820	D86194
94	*B. pseudolongum* subsp. *pseudolongum*	DSM 20099 = LMG 11571	NZ_JDTZ01000002	JCM 1205	LC071797
95	*B. psychraerophilum*	DSM 22366	NZ_JDUQ01000014	T16	NR_029065
96	*B. psychraerophilum*	LMG 21775	JGZI01000009	YIT 11814	AB437351
97	*B. pullorum*	DSM 20433 = JCM 1214	NZ_JDUI01000001	JCM 1214	D86196
98	*B. reuteri*	DSM 23975	NZ_JDUW01000002	DSM 23975	NZ_JDUW01000049
99	*B. ruminantium*	DSM 6489 = JCM 8222	NZ_JHWQ01000003	JCM 8222	D86197
100	*B. ruminantium*	LMG 21811	JGZL01000008	LMG 21811	JGZL01000002
101	*B. saeculare*	DSM 6531	JGZM01000001	DSM 6531	D89328
102	*B. saguini*	DSM 23967	JGZN01000006	DSM 23967	AB559504
103	*B. scardovii*	JCM 12489 = DSM 13734	AP012331	DSM 13734	AP012331
104	*B. scardovii*	LMG 21589	JGZO01000003	LMG 21589	JGZO01000008
105	*B. stellenboschense*	DSM 23968	NZ_JGZP01000019	DSM 23968	JGZP01000012
106	*B. subtile*	DSM 20096	NZ_AUFH01000005	DSM 20096	D89378
107	*B. subtile*	LMG 11597	JGZR01000002	LMG 11597	JGZR01000006
108	*B. thermacidophilum subsp. porcinum*	LMG 21689	JGZS01000003	LMG 21689	JGZS01000003
109	*B. thermacidophilum* subsp. *thermacidophilum*	DSM 15837	NZ_AUFI01000014	YIT 11849	AB437362
110	*B. thermacidophilum* subsp. *thermacidophilum*	LMG 21395	JGZT01000008	LMG 21395	JGZT01000008
111	*B. thermophilum*	DSM 20210 = ATCC 25525	NZ_JDUB01000001	ATCC 25525	U10151
112	*B. thermophilum*	RBL67	CP004346	RBL67	DQ340557
113	*B. tsurumiense*	DSM 17777 = OMB115	NZ_AUCL01000007	OMB115	AB241106
114	*B. tsurumiense*	JCM 13495	JGZU01000007	JCM 13495	LC258148
115	*B. aquikefiri*	LMG 28769	NZ_MWXA01000008	LMG 28769	NR_148810
116	*B. bombi*	DSM 19703	NZ_ATLK01000001	DSM 19703	ATLK01000001
117	*B. hapali*	DSM 100202	NZ_MWWY01000021	MRM_9.14	KP718963
118	*B. myosotis*	DSM 100196	NZ_MWWW01000006	MRM_5.10	KP718942
119	*B. tissieri*	DSM 100201	NZ_MWWV01000004	DSM 100201	MWWV01000031
120	*B. vansinderenii*	Tam10B	NEWD01000003	Tam10B	NZ_NEWD01000058

[a] ATCC, American Type Culture Collection; DSM, Deutsche Sammlung von Mikroorganismen und Zellkulturen; JCM, Japanese Collection of Microorganisms; LMG, Laboratorium voor Microbiologie, University of Ghent.

Table S2. Quantification of *Bifidobacterium* species in human feces.

Sample ID	Log10 bifidobacteria/g of feces (wet weight)		
	**Total bifidobacteria**	***B. pseudocatenulatum***	***B. longum* subsp. *longum***
H1	9.03	8.10	8.48
H2	9.24	8.94	8.64
H3	9.58	9.42	8.88
H4	9.17	8.77	8.58
H5	9.83	9.40	9.52
H6	9.41	9.06	8.84
H7	9.62	9.36	8.76
H8	9.09	8.92	8.09

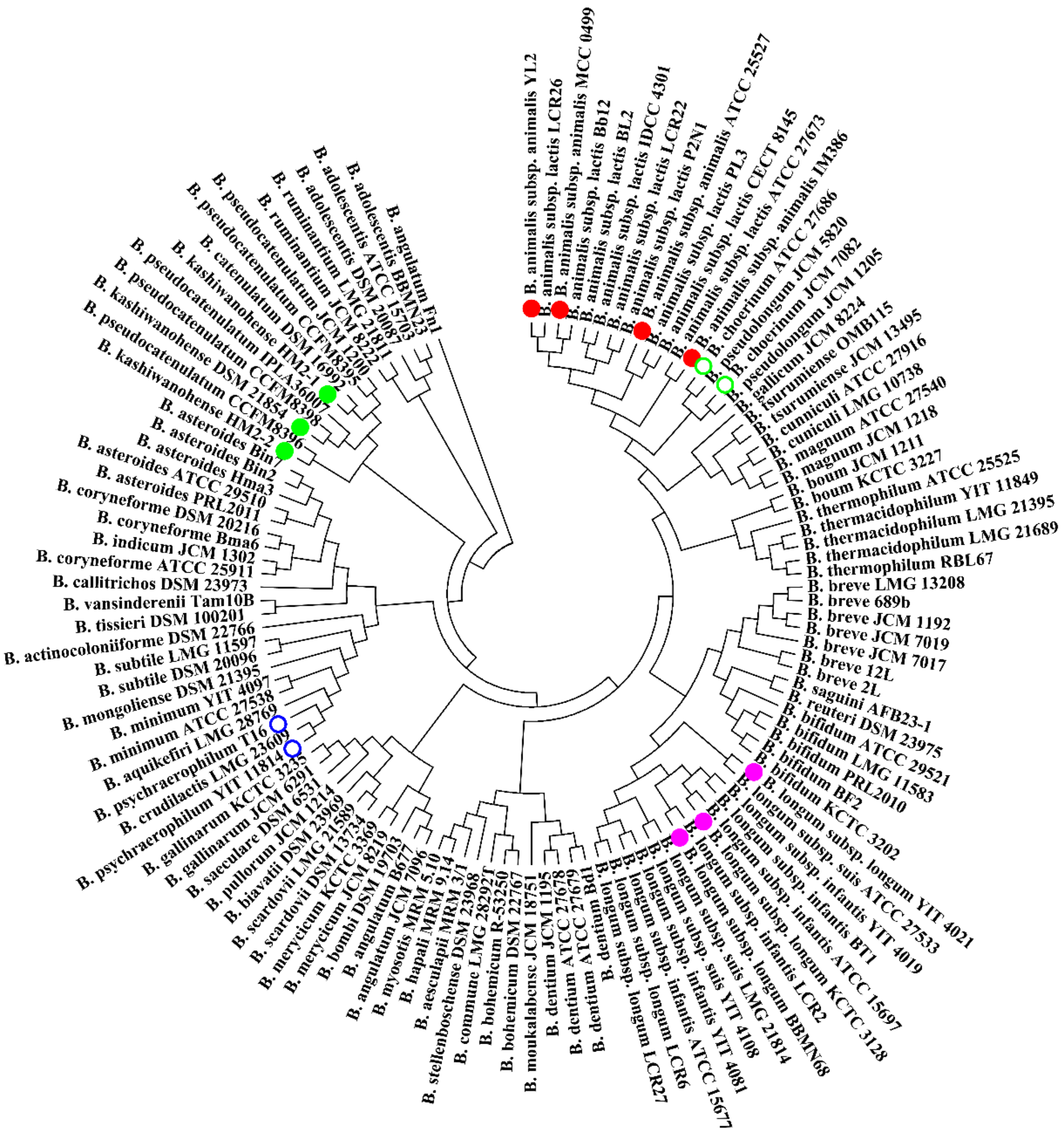

Figure S1. Phylogenetic tree based on the V3–V4 region sequences of the 16S rRNA gene. The tree was constructed by Maximum likelihood method with bootstrap values calculated from 1000 trees.
